# MicroRNAs as possible indicators of drug sensitivity in breast cancer cell lines

**DOI:** 10.1371/journal.pone.0216400

**Published:** 2019-05-07

**Authors:** Katharina Uhr, Wendy J. C. Prager-van der Smissen, Anouk A. J. Heine, Bahar Ozturk, Marijn T. M. van Jaarsveld, Antonius W. M. Boersma, Agnes Jager, Erik A. C. Wiemer, Marcel Smid, John A. Foekens, John W. M. Martens

**Affiliations:** Department of Medical Oncology and Cancer Genomics Netherlands, Erasmus MC Cancer Institute, Erasmus University Medical Center, Rotterdam, The Netherlands; Virginia Commonwealth University, UNITED STATES

## Abstract

MicroRNAs (miRNAs) regulate gene expression post-transcriptionally. In this way they might influence whether a cell is sensitive or resistant to a certain drug. So far, only a limited number of relatively small scale studies comprising few cell lines and/or drugs have been performed. To obtain a broader view on miRNAs and their association with drug response, we investigated the expression levels of 411 miRNAs in relation to drug sensitivity in 36 breast cancer cell lines. For this purpose IC_50_ values of a drug screen involving 34 drugs were associated with miRNA expression data of the same breast cancer cell lines. Since molecular subtype of the breast cancer cell lines is considered a confounding factor in drug association studies, multivariate analysis taking subtype into account was performed on significant miRNA-drug associations which retained 13 associations. These associations consisted of 11 different miRNAs and eight different drugs (among which Paclitaxel, Docetaxel and Veliparib). The taxanes, Paclitaxel and Docetaxel, were the only drugs having miRNAs in common: *hsa-miR-187-5p* and *hsa-miR-106a-3p* indicative of drug resistance while Paclitaxel sensitivity alone associated with *hsa-miR-556-5p*. Tivantinib was associated with *hsa-let-7d-5p* and *hsa-miR-18a-5p* for sensitivity and *hsa-miR-637* for resistance. Drug sensitivity was associated with *hsa-let-7a-5p* for Bortezomib, *hsa-miR-135a-3p* for JNJ-707 and *hsa-miR-185-3p* for Panobinostat. Drug resistance was associated with *hsa-miR-182-5p* for Veliparib and *hsa-miR-629-5p* for Tipifarnib. Pathway analysis for significant miRNAs was performed to reveal biological roles, aiding to find a potential mechanistic link for the observed associations with drug response. By doing so *hsa-miR-187-5p* was linked to the cell cycle G2-M checkpoint in line with this checkpoint being the target of taxanes. In conclusion, our study shows that miRNAs could potentially serve as biomarkers for intrinsic drug resistance and that pathway analyses can provide additional information in this context.

## 1. Introduction

Biomarkers of drug sensitivity/resistance are of great interest for the clinic as they allow for patient stratification–thereby identifying the most effective therapy for patients faster, reducing overtreatment and toxicity burden as well as saving costs. In the search for highly suited biomarkers irrespective of research field or application, microRNAs (miRNAs) have become increasingly popular due to their stability and broad applicability, underlining their potential as biomarkers [1,2].

MiRNAs are small oligonucleotides involved in multiple processes such as aging, tissue development and also cancer [3–5]. They bind in a sequence-dependent fashion to mRNA 3’ UTRs leading to inhibition of messenger RNA (mRNA) translation, endonucleolytic cleavage of the mRNA or mRNA destabilization. Through affecting mRNA translation and stability, miRNAs ultimately regulate protein expression [6,7]. As miRNA-recognition sequences are present in many genes, one miRNA can impact up to several hundred transcripts [7–12].

Until now, several studies have been conducted to link miRNAs to treatment outcome [13,14]. In these earlier studies, mainly drug-resistant cell lines were generated, which were then compared to their parental cell lines to reveal differentially expressed miRNAs, which were subsequently studied in functional experiments. While these experiments are instrumental for a mechanistic understanding of miRNAs in drug sensitivity/resistance, such experiments do not necessarily identify predictors of drug resistance or sensitivity. Furthermore, most of these studies involved only one drug and were performed with a limited number of cell lines. As breast tumors can be classified into distinct subtypes with different clinical outcome [15,16] this should also be reflected in study design by including larger series of cell lines, together better resembling the clinical variability. Furthermore, screening for drug resistance against many drugs may not only identify miRNAs associated with one compound but may also identify miRNAs linked to sensitivity/resistance of more than one drug.

Therefore, we analyzed 36 well-characterized breast cancer cell lines that we screened for sensitivity to 34 compounds to obtain more clinically meaningful results on miRNAs and their potential as biomarkers for drug response in breast cancer. Our study showed that this approach was feasible and led to the identification of several miRNA-drug associations not found earlier.

## 2. Materials and methods

### 2.1 Breast cancer cell lines

Cell lines were cultured and profiled for correct identity as described previously [17]. In summary: Cell lines were cultured for RNA isolation until 70–80% confluence on collagen-coated petri dishes in triplicate. RPMI 1640 medium with 10% heat-inactivated fetal bovine serum and antibiotic agents (80 μg/ml (0.08 kg/m^3^) Streptomycin and 100 μg/ml (0.1 kg/m^3^) Penicillin G) was used as medium.

To ensure cell line identity, DNA of the 36 breast cancer cell lines was isolated using the QIAamp DNA Mini Kit (Qiagen, Hilden, Germany) and subsequently subjected to short-tandem repeat (STR) profiling using the PowerPlex16 system (Promega, Madison, WI, USA) following manufacturer’s protocol. A 3130xl Genetic Analyzer (Applied Biosystems, Foster City, CA, USA) was used for detection and Genemarker 1.91 software from Softgenetics (State College, PA, USA) was employed for analysis. STR profiles were compared with those deposited at the American Type Culture Collection (Manassas, VA, USA) and the Deutsche Sammlung von Mikroorganismen und Zellkulturen (Braunschweig, Germany). For SUM cell lines, STR profiles were not available, so in-house profiles from the earliest passages were compared with those generated later. Cell lines had been obtained between 1997 and 2006 from the following sources:

American Type Culture Collection (BT-20 [18], BT-474 [19], BT-549 [20], CAMA-1 [21], HCC1143 [22], HCC1395 [22], HCC1569 [23], HCC1806 [23], HCC-1937 [24], HCC1954 [22], HCC202 [22], HCC38 [25], HCC70 [22], Hs578T [26], MCF-7 [27], MDA-MB-175VII [28], MDA-MB-231 [28], MDA-MB-361 [28], MDA-MB-415 [29], MDA-MB-436 [29], MDA-MB-468 [29], SK-BR-3 [30], T47D [31], UACC812 [32]),

Dr. N. de Vleesschouwer (Institut Jules Bordet, Brussels, Belgium) (EVSA-T [33]),

Dr. H.S. Smith (California Pacific Medical Center, San Francisco, CA, USA) (MPE-600 [34]),

Dr. E. Stockert (Sloan-Kettering Institute for Cancer Research, New York, NY, USA) (SK-BR-7 [35]),

Ethier laboratory (BioIVT, West Sussex, UK) (SUM1315M02 [36], SUM149PT [36], SUM159PT [36], SUM185PE [36], SUM190PT [36], SUM229PE [36], SUM44PE [37], SUM52PE [38]), Riken Gene Bank (Tsukuba, Japan) (OCUB-M [39])

### 2.2 Drug screening

Drug screening data was previously published for an extended cell line panel and the detailed description of drug-drug relations as well as the entire drug response dataset is available there [40]. In brief: The breast cancer cell lines were cultured and plated in triplicate in 96-well collagen-coated plates, and then incubated for 96 hours with 12 serial dilutions of 37 drugs or vehicle per cell line. The drugs were supplied by Janssen Pharmaceutica (Beerse, Belgium) except for Veliparib, which was supplied by Abbott Laboratories (North Chicago, IL, USA). After 96 hours the Sulforhodamine B Assay was used to quantify total protein as a measure of cell number [41] and IC_50_ values were computed subsequently [40]. Three drugs did not exhibit differential responses among the cell lines and were excluded from the final dataset [40]. Therefore, the present study included 34 drugs which were used for further analyses.

### 2.3 RNA isolation

RNA isolation was performed as described earlier [17]. To summarize: RNAzol-B reagent (Campro Scientific BV, Veenendaal, the Netherlands) was used for isolation of total RNA from all cell lines according to manufacturer’s protocol. Quality of isolated RNA was verified using spectrophotometric assessments of the A_260nm_/A_280 nm_ and the A_260nm_/A_230nm_ ratios, with the first required to have a value of approximately 2 and the second to have a value of 2 or higher. For these measurements a NanoDrop ND-1000 (Isogen Life Science, De Meern, the Netherlands) was used. Additional quality checks were performed as described earlier [42].

### 2.4 Gene expression profiling

Gene expression profiling was performed as previously described [17]. Reverse transcription was performed on 200 ng (2E-10 kg) of total RNA, followed by cDNA synthesis and the generation of biotin-labeled cRNA using the 3’ IVT express kit (Affymetrix, Santa Clara, CA, USA). Fragmentation of the labeled cRNA ensued and finally the fragmented and labeled cRNA was loaded on the Affymetrix GeneTitan. The hybridization mixture was loaded on Affymetrix Human_Genome_HT_HG-U133_Plus_PM GeneChip 96-well arrays. The subsequent steps (hybridization, washing and scanning) were performed within the GeneTitan. In the Affymetrix Expression Console the generated “.CEL” files were subjected to normalization employing the default settings of the RMA method. The generated data have been uploaded to the Gene Expression Omnibus data repository under the following access code: [GEO:GSE41313].

Breast cancer subtypes were determined using hierarchical clustering. Clusters were obtained with average distance linkage hierarchical clustering with non-centered correlation as distance metric. The three resulting clusters were matched to previously established intrinsic subtypes [43] of these cell lines (which were based on a different chip-type) and the clusters were labeled as ‘basal’, ‘luminal’ and ‘normal-like’.

### 2.5 miRNA expression profiling

The procedure employed for miRNA expression profiling has been described previously in detail [17,44]. MiRNA expression data of cell lines with confirmed identity were used. To generate the data set, one microgram (1E-09 kg) of total RNA was labeled with the dye Cy3 using the ULS aRNA labeling kit (Kreatech, Amsterdam, the Netherlands). Only RNA samples with a labeling efficiency higher than 15 pmol Cy3/μg RNA (1.5E-20 mol/kg) were used for hybridization. One sample was used per cell line. Hybridization was performed overnight. Normalization of the data was performed as previously described [44]. Labeled total RNA isolated from all cell lines was hybridized to home-made microarrays containing LNA modified capture probes from Exiqon (Vedbaek, Denmark) for the miRNA capture. This miRNA microarray design was based on miRBase version 10.0 (annotation version 13), contained 1344 probes and had the capacity to measure the expression levels of 725 human miRNAs.

Within this dataset two miRNAs (*hsa-miR-185* and *hsa-miR-620*) were represented twice on the microarray with differential probes. We removed the measurements with the older probes (*hsa-miR-185* probe ID 5560 and *hsa-miR-620* probe ID 32825) and kept the measurements of the newer probes (miRNA *hsa-miR-185* probe ID 42902 and miRNA *hsa-miR-620* probe ID 42994). We assessed whether the probe version influenced results, however, this was not the case.

The removal of the older probes resulted in a final number of 411 miRNAs expressed above background. MiRNA expression data has been published earlier [17], except for the cell line OCUB-M, the miRNA expression data for this cell line can be found in **S1 Table**. The significant miRNAs are listed with the Exiqon oligonucleotide ID and the different miRBase [45] annotations, as well the corresponding MIMAT identifier in **S2 Table**.

### 2.6 Association of miRNA expression with IC_50_ values and co-expression analysis

By inspecting the results of the drug screening it became evident that the IC_50_ response curves for different drugs were very different across cell lines (see also Uhr *et al*.[40]). While for some drugs most cell lines had different IC_50_ values, other drugs had a large number of cell lines with the same IC_50_ value up to almost all cell lines having identical IC_50_ values. Due to this it became clear that different analyses would be required to account for the different IC_50_ drug profiles. For most drugs, several cell lines had an IC_50_ value which was at the maximum of the tested drug concentration. Depending on the number of cell lines with values at maximum drug level, we chose to either use Spearman correlation (≤ 5 maximum IC_50_ values), a 2-step analysis (>5 IC_50_ values at maximum & ≥10 IC_50_ values not at the maximum) or a Mann-Whitney test (<10 IC_50_ values which are not at the maximum). MiRNA data were log10 –transformed for analyses, drug data were transformed to negative log 10 values.

The first step of the 2-step analysis consisted of Spearman correlation of those IC_50_ values that were not at the maximum drug concentration (“variable IC_50_ values”) with miRNA expression values. Identified miRNA-drug associations were then tested in the second step, a Mann-Whitney test, classifying all IC_50_ values at maximum drug concentration as resistant and the remaining variable IC_50_ values as sensitive.

Over the results of each analysis group (Spearman correlation, 2-step analysis, Mann-Whitney test), q-values [46] were calculated to account for multiple testing. A q-value of 0.3 or lower was determined significant. The analysis type used per drug is listed in **S3 Table**.

Analyses were performed in R (versions 3.4.4 up to 3.5.1) [47] using the psych package [48] and corr.test function for Spearman correlations, setting alpha at 0.05. The R function wilcox.test was used for Mann-Whitney testing. Q-values were calculated using the q value Bioconductor package [49] in R.

**Fig 1:**
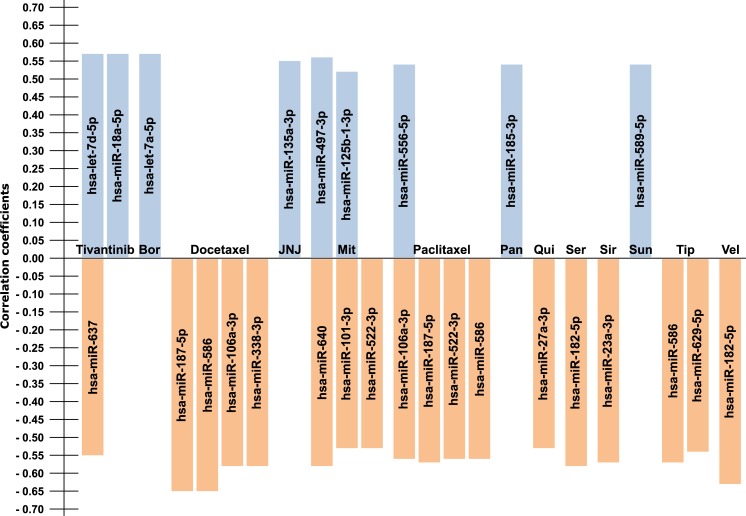
Overview of miRNAs associated with different drugs. The correlation coefficient is given on the y-axis. Associations with drug resistance are given in orange; associations with drug sensitivity are given in blue. Bor = Bortezomib, JNJ = JNJ-707, Mit = Mitoxantrone, Pan = Panobinostat, Qui = Quisinostat, Ser = Serdemetan, Sir = Sirolimus, Sun = Sunitinib, Tip = Tipifarnib, Vel = Veliparib.

Fig 1 was created in Inkscape 0.92 (Free Software Foundation Inc., Boston, USA).

### 2.7 Multivariate analysis

MiRNAs significantly associated with drug response were evaluated in a multivariate analysis, including gene-expression-based breast cancer subtype as a co-variate. Linear regression was used, as only drugs with a linear IC_50_ profile had shown significant associations with miRNA expression. Subtypes were used as a binary variable: basal vs rest, luminal vs rest and normal-like vs rest. The IC_50_ values were used as the dependent variable, while the miRNA levels as well as one of the binary subtype groups were used as independent variables. This regression model was run for each of the three binary subtype-groups separately and was calculated using Stata—version 13 (StataCorp, College Station, TX, USA).

### 2.8 Pathway analyses

Pathway analyses were performed on the mRNA data described above. The 11 significant miRNAs that were not related to subtype were analyzed by grouping the cell lines into top and bottom 25% based on the expression of the respective miRNA. This led to 9 cell lines per group. The mRNA expression of top and bottom cell lines was analyzed for differences in R [47] using the Global Test package [50] and Biocarta (San Diego, CA, USA) and KEGG [51] pathway information. P-values were subjected to multiple testing correction (method “BH”, also known as “FDR”) and a permutation test (1000 permutations) of the non-adjusted p-value was used as an additional selection criterion. Associations with BH-adjusted p-values below 0.1 and permutation p-values below 0.05 were deemed significant.

## 3. Results

### 3.1 Association of miRNAs with drug sensitivity

In the analysis we combined the data of 36 cell lines for which 34 drugs gave differential responses among the included cell lines [40] and the expression data of 411 miRNAs [17].

To associate miRNA expression with drug response, we first inspected the IC_50_ profiles of all 34 compounds among the breast cancer cell lines. Based on the number of cell lines with a maximum IC_50_ value we chose one of three different analysis approaches as defined in Materials and Methods.

Only the drugs with “linear” IC_50_ values showed significant associations with miRNA expression upon q-value correction, totaling to 27 miRNA-drug associations (see **Table 1 and Fig 1**).

**Table 1:** MiRNAs associated with drug response.

Drug	MiRNA	Association type	R	p-value	q-value
Tivantinib	*hsa-let-7d-5p*	Sensitivity	0.57	3.96E-04	0.18
Tivantinib	*hsa-miR-18a-5p*	Sensitivity	0.57	3.88E-04	0.18
Tivantinib	*hsa-miR-637*	Resistance	-0.55	6.51E-04	0.24
Bortezomib	*hsa-let-7a-5p*	Sensitivity	0.57	2.94E-04	0.18
Docetaxel	*hsa-miR-187-5p*	Resistance	-0.65	1.89E-05	0.07
Docetaxel	*hsa-miR-586*	Resistance	-0.65	1.99E-05	0.07
Docetaxel	*hsa-miR-106a-3p*	Resistance	-0.58	2.26E-04	0.18
Docetaxel	*hsa-miR-338-3p*	Resistance	-0.58	1.85E-04	0.18
JNJ-707	*hsa-miR-135a-3p*	Sensitivity	0.55	5.15E-04	0.21
Mitoxantrone	*hsa-miR-497-3p*	Sensitivity	0.56	3.83E-04	0.18
Mitoxantrone	*hsa-miR-640*	Resistance	-0.58	1.95E-04	0.18
Mitoxantrone	*hsa-miR-101-3p*	Resistance	-0.53	8.85E-04	0.27
Mitoxantrone	*hsa-miR-522-3p*	Resistance	-0.53	9.67E-04	0.27
Mitoxantrone	*hsa-miR-125b-1-3p*	Sensitivity	0.52	1.07E-03	0.29
Paclitaxel	*hsa-miR-106a-3p*	Resistance	-0.56	4.17E-04	0.18
Paclitaxel	*hsa-miR-187-5p*	Resistance	-0.57	2.64E-04	0.18
Paclitaxel	*hsa-miR-522-3p*	Resistance	-0.56	3.44E-04	0.18
Paclitaxel	*hsa-miR-586*	Resistance	-0.56	3.40E-04	0.18
Paclitaxel	*hsa-miR-556-5p*	Sensitivity	0.54	7.65E-04	0.26
Panobinostat	*hsa-miR-185-3p*	Sensitivity	0.54	6.47E-04	0.24
Quisinostat	*hsa-miR-27a-3p*	Resistance	-0.53	9.06E-04	0.27
Serdemetan	*hsa-miR-182-5p*	Resistance	-0.58	2.08E-04	0.18
Sirolimus	*hsa-miR-23a-3p*	Resistance	-0.57	2.46E-04	0.18
Sunitinib	*hsa-miR-589-5p*	Sensitivity	0.54	7.35E-04	0.26
Tipifarnib	*hsa-miR-586*	Resistance	-0.57	3.54E-04	0.18
Tipifarnib	*hsa-miR-629-5p*	Resistance	-0.54	9.01E-04	0.27
Veliparib	*hsa-miR-182-5p*	Resistance	-0.63	4.25E-05	0.1

MiRNAs significantly associated with drug response. Associations were computed using Spearman’s correlation and the correlation coefficient (R) is given, next to the unadjusted p-value and the q-value.

### 3.2 Breast cancer subtype as potential confounder of miRNA drug associations

In a previous study [40] we had found that certain breast cancer subtypes can respond differently to some drugs. As expression of certain miRNAs might be related to a specific subtype, we assessed our significant findings in a multivariate analysis. In this analysis we modeled the association between drug response and miRNA expression and subtype. All 27 associations were tested with subtype and the respective results are listed in **S4 Table**. Fourteen miRNA-drug associations were linked to one or more breast cancer subtypes. Basal subtype was associated stronger with the respective miRNA than the associated drug for the following associations: *hsa-miR-586* and Paclitaxel, *hsa-miR-589-5p* and Sunitinib and *hsa-miR-586* and Tipifarnib. Luminal subtype affected the association between *hsa-miR-27a-3p* and Quisinostat as well as *hsa-miR-23a-3p* and Sirolimus. Normal-like subtype confounded the associations between *hsa-miR-101-3p* and Mitoxantrone, *hsa-miR-125b-1-3p* and Mitoxantrone, *hsa-miR-497-3p* and Mitoxantrone, *hsa-miR-640* and Mitoxantrone and *hsa-miR-522-3p* and Paclitaxel. Several associations were related to more than one subtype: These were the subtypes basal and luminal for the two Docetaxel-associated miRNAs *hsa-miR-338-3p* and *hsa-miR-586*. Furthermore, the subtypes luminal and normal-like showed an effect on the association between *hsa-miR-522-3p* and Mitoxantrone and the subtypes basal and normal-like were implicated in the association between *hsa-miR-182-5p* and Serdemetan.

Molecular subtype did not affect the 13 remaining miRNA-drug associations (8 drugs, 11 miRNAs), which are highlighted in more detail below.

Sensitivity to the c-Met inhibitor Tivantinib [52] was associated with the expression of two miRNAs, i.e. *hsa-let-7d-5p* and *hsa-miR-18a-5p*, while drug resistance was associated with *hsa-miR-637*. Drug sensitivity to the proteasome inhibitor Bortezomib [53] was associated with *hsa-let-7a-5p* expression. Among the microtubule-targeting taxanes Docetaxel and Paclitaxel [54], we found that *hsa-miR-106a-3p* and *hsa-miR-187-5p* expression were positively correlated with drug resistance for both drugs. *Hsa-miR-556-5p* expression, however, was solely associated with Paclitaxel sensitivity. *Hsa-miR-135a-3p* expression was related to sensitivity to JNJ-707, a drug targeting FGF receptors [40]. Panobinostat, one of the HDAC inhibitors [55] in our screening, showed a positive correlation with *hsa-miR-185-3p* expression for drug sensitivity. For Tipifarnib, a farnesyltransferase inhibitor targeting RAS signal transduction [56], expression of *hsa-miR-629-5p* was associated with resistance to the drug, while drug resistance to the PARP inhibitor Veliparib [57] was associated with *hsa-miR-182-5p* expression.

Besides the confounding effect of the breast cancer subtypes on some miRNA-drug associations, we also noted that several drugs had more than one associated miRNA, which led to our next analysis whether miRNAs are co-expressed.

### 3.3 Co-expression among miRNAs associated with the same drug

To study whether miRNAs that significantly associated with the same drug in univariate analysis showed a correlated expression pattern, we performed a correlation analysis. Below we discuss Tivantinib, Docetaxel and Paclitaxel, as each of these three drugs had several associated miRNAs unrelated to one of the breast cancer subtypes (see **S4 Table**). Correlation coefficients and p-values are given in the **S5–S7 Tables**. In the case of Tivantinib the miRNAs *hsa-miR-18a-5p* and *hsa-let-7d-5p* showed a mild positive correlation with each other (R = 0.28), which was however not significant, while *hsa-miR-637* showed a significant negative association with the aforementioned miRNAs (R = -0.49 and -0.46, respectively). The two Docetaxel- and Paclitaxel-associated miRNAs *hsa-miR-187-5p* and *hsa-miR-106a-3p* showed an intermediate significant positive correlation with each other (R = 0.40), while the other Paclitaxel-associated miRNA *hsa-miR-556-5p* was significantly negatively associated with *hsa-miR-187-5p* and *hsa-miR-106a-3p* (R = -0.50 and -0.44, respectively).

### 3.4 Pathway analyses on significant miRNAs

In the next step, we performed pathway analyses to learn more about the biology of the miRNAs associated with drug response in the breast cancer cell lines. For this purpose we used those 11 miRNAs which were significantly associated with drug response independent of subtypes (see **Table 1** and **S4 Table**). Messenger RNA expression data from the cell lines with the most differential expression of each miRNA (high versus low expression were compared; 9 per subgroup) were used as input for the pathway analysis. Pathways which were significantly different between samples with high or low expression of a tested miRNA are shown in **Table 2**.

**Table 2:** Pathways associated with miRNAs.

MiRNA	Pathway	Permutation p-value	BH-adjusted p-vaule pp-value	Associated drugs
*hsa-miR-106a-3p*	TPO Signaling Pathway (Biocarta)	1.00E-03	7.48E-02	Docetaxel, Paclitaxel
* *	Nerve growth factor pathway NGF (Biocarta)	1.00E-03	7.48E-02	
* *	T Cell Receptor Signaling Pathway (Biocarta)	0.00E+00	9.74E-02	
* *	Insulin Signaling Pathway (Biocarta)	2.00E-03	9.74E-02	
* *	Signaling Pathway from GProtein Families (Biocarta)	3.00E-03	9.74E-02	
* *	Cadmium induces DNA synthesis and proliferation in macrophages (Biocarta)	3.00E-03	9.74E-02	
* *	EPO Signaling Pathway (Biocarta)	6.00E-03	9.74E-02	
*hsa-miR-135a-3p*	Pantothenate and CoA biosynthesis (KEGG)	0.00E+00	5.39E-02	JNJ-707
*hsa-miR-182-5p*	Intrinsic Prothrombin Activation Pathway (Biocarta)	4.00E-03	5.02E-02	Veliparib
* *	Role of Tob in Tcell activation (Biocarta)	8.00E-03	5.02E-02	
* *	Acute Myocardial Infarction (Biocarta)	1.20E-02	5.02E-02	
* *	Actions of Nitric Oxide in the Heart (Biocarta)	1.20E-02	5.02E-02	
* *	Erythrocyte Differentiation Pathway (Biocarta)	1.30E-02	5.02E-02	
* *	Signal transduction through IL1R (Biocarta)	1.70E-02	5.02E-02	
* *	ALK in cardiac myocytes (Biocarta)	1.70E-02	5.02E-02	
* *	Cytokines and Inflammatory Response (Biocarta)	1.90E-02	5.02E-02	
* *	Integrin Signaling Pathway (Biocarta)	2.30E-02	5.02E-02	
* *	Estrogenresponsive protein Efp controls cell cycle and breast tumors growth (Biocarta)	3.00E-02	5.02E-02	
* *	Selective expression of chemokine receptors during Tcell polarization (Biocarta)	3.30E-02	5.02E-02	
* *	Cell Cycle G1 S Check Point (Biocarta)	3.30E-02	5.02E-02	
* *	Cytokine Network (Biocarta)	3.30E-02	5.58E-02	
* *	Phosphoinositides and their downstream targets (Biocarta)	4.10E-02	5.02E-02	
* *	Role of ERBB2 in Signal Transduction and Oncology (Biocarta)	4.30E-02	5.02E-02	
* *	Hypoxia and p53 in the Cardiovascular system (Biocarta)	4.40E-02	5.36E-02	
* *	Trefoil Factors Initiate Mucosal Healing (Biocarta)	4.70E-02	6.71E-02	
* *	TGFbeta signaling pathway (KEGG)	8.00E-03	8.41E-02	
* *	Focal adhesion (KEGG)	1.30E-02	8.41E-02	
*hsa-miR-187-5p*	Selective expression of chemokine receptors during Tcell polarization (Biocarta)	3.00E-03	7.70E-02	Docetaxel, Paclitaxel
* *	Trefoil Factors Initiate Mucosal Healing (Biocarta)	3.00E-03	7.70E-02	
* *	CXCR4 Signaling Pathway (Biocarta)	1.60E-02	7.70E-02	
* *	Regulation of eIF4e and p70 S6 Kinase (Biocarta)	1.80E-02	7.70E-02	
* *	Skeletal muscle hypertrophy is regulated via AKT mTOR pathway (Biocarta)	2.00E-02	7.70E-02	
* *	CARM1 and Regulation of the Estrogen Receptor (Biocarta)	2.10E-02	7.70E-02	
* *	Neuropeptides VIP and PACAP inhibit the apoptosis of activated T cells (Biocarta)	2.30E-02	7.70E-02	
* *	Estrogenresponsive protein Efp controls cell cycle and breast tumors growth (Biocarta)	2.80E-02	7.70E-02	
* *	Role of BRCA1 BRCA2 and ATR in Cancer Susceptibility (Biocarta)	2.80E-02	7.70E-02	
* *	Role of ERBB2 in Signal Transduction and Oncology (Biocarta)	3.30E-02	7.70E-02	
* *	Role of Tob in Tcell activation (Biocarta)	3.30E-02	8.44E-02	
* *	Cell Cycle G2 M Checkpoint (Biocarta)	3.50E-02	7.70E-02	
* *	mTOR Signaling Pathway (Biocarta)	3.90E-02	7.70E-02	
* *	Effects of calcineurin in Keratinocyte Differentiation (Biocarta)	4.20E-02	7.70E-02	
* *	Nitrogen metabolism (KEGG)	3.00E-03	2.56E-02	
* *	AminoacyltRNA biosynthesis (KEGG)	3.00E-03	2.77E-02	
* *	Valine leucine and isoleucine degradation (KEGG)	1.90E-02	3.35E-02	
* *	Pentose phosphate pathway (KEGG)	6.00E-03	4.87E-02	
* *	Wnt signaling pathway (KEGG)	4.80E-02	8.28E-02	
*hsa-miR-556-5p*	Signaling Pathway from GProtein Families (Biocarta)	1.00E-03	4.86E-02	Paclitaxel
*hsa-miR-637*	Links between Pyk2 and Map Kinases (Biocarta)	1.00E-03	5.46E-02	Tivantinib

Pathways significantly associated with drug-associated miRNAs. Selection criteria for significant pathways were a permutation p-value below 0.05 and a Benjamini-Hochberg adjusted p-value below 0.1. The respective associated drugs are listed and highlighted in orange for an association of the miRNA with resistance to the respective drug and blue for an association with sensitivity.

For 6 of the 11 miRNAs we found significantly associated pathways. The miRNAs *hsa-miR-135a-3p*, *hsa-miR-556-5p and hsa-miR-637* each had only 1 pathway associated, while *hsa-miR-106a-3p* had 7 pathways associated and *hsa-miR-182-5p* and *hsa-miR-187-5p* each had 19 pathways significantly associated. For the 3 miRNAs associated with multiple pathways, these pathways typically included the same genes such as in the case of *hsa-miR-106a-3p* with the genes *JUN* and *FOS* upregulated in all significant pathways and *PIK3R1* being upregulated in 3 of the 7 pathways. In the case of *hsa-miR-182-5p* many of the associated pathways had genes of the COL4A family and the TGF-β family downregulated, while for *hsa-miR-187-5p* several of the pathways contained the genes *CXCR4*, *ESR1*, *PDK2*, *PTEN*; as well as the ER-regulated genes *TFF1* [58] and *TFF3* [59].

## 4. Discussion

Our exploratory investigation showed that the expression of specific miRNAs can indicate drug sensitivity or resistance in breast cancer cell lines. In total, we were able to identify 21 different miRNAs associated with response to 13 different drugs, totaling up to 27 miRNA-drug associations. After excluding miRNAs that also associate with breast cancer subtype in multivariate analyses, 11 miRNAs involved in 13 miRNA-drug associations remained. Additional pathway analyses using available gene expression data gave insights into the associated biology for 6 of the significant miRNAs. To assess the applicability of these miRNAs as clinical biomarkers, further validation in independent sample series is needed to correct for possible additional parameters influencing drug response which were not considered here. Separately, the causal relation between the identified miRNA-drug associations in this hypothesis-generating study could be explored.

To try to understand the subtype independent miRNA-drug associations we discuss the possible mechanistic links for the most significant results based on current state-of-the-art knowledge.

The strongest miRNA-drug interaction was the association of *hsa-miR-187-5p* with Docetaxel resistance. This miRNA was additionally associated with Paclitaxel resistance. As taxanes have been known for a long time for their anti-tumorigenic activity and have been studied extensively [60], several resistance mechanisms are known: One of the main contributors to taxane resistance is the overexpression of multidrug transporters (e.g. P-gp; HGNC symbol: *ABCB1*) [61]. Besides, tubulin modifications (e.g. mutations) and alterations in the tubulin-microtubule system have been found such as altered expression of tubulin isoforms or associated proteins (e.g. MAP4 and Stathmin) [61]. While we did not find a clear link between *hsa-miR-187-5p* and one of these well-known resistance mechanisms, we found that this miRNA is associated with the pathway “Cell cycle G2-M checkpoint” and the “MTOR signaling” pathway (see **Table 2**). This is an interesting observation, as Paclitaxel is known to block cells between the G2 and the mitotic phase of the cell cycle [60]. Furthermore, genes involved in the G2-M checkpoint and MTOR signaling were differentially expressed in residual breast tumors after Docetaxel treatment when compared to pre-treatment biopsies [62]. In our study the genes in the G2-M checkpoint pathway are mainly downregulated in cell lines with high *hsa-miR-187-5p* expression, arguing that this miRNA might target several of the involved genes or an upstream regulator and in this way potentially increases cell cycle progression. Alternatively, *hsa-miR-187-5p* could be co-regulated with the genes in this pathway and as a result might affect events downstream.

The miRNA *hsa-miR-106a-3p* was also associated with Docetaxel and Paclitaxel resistance. In the pathway analysis, 7 pathways were significantly associated with this miRNA. Looking further into similarities between the pathways, it became clear that the genes *JUN* and *FOS* were part of all pathways and were clearly upregulated. Unfortunately only little is known about this miRNA and no link could be found between either the genes *JUN* and *FOS* or this miRNA with resistance to the two taxanes Docetaxel and Paclitaxel. As the expression of this miRNA is correlated to the expression of *hsa-miR-187-5p* in our study, this miRNA could act in concert with *hsa-miR-187-5p* or its associated pathways but based on current knowledge the role of this miRNA in taxane resistance remains to be clarified at this stage.

Within our drug screening, we had one PARP inhibitor, Veliparib [63] and we observed that *hsa-miR-182-5p* was associated with resistance to this drug. This finding contrasts an earlier study in which *hsa-miR-182-5p* was found to target *BRCA1* and sensitized the breast cancer cell line MDA-MB-231 to PARP inhibitors [64]. Neijenhuis *et al*. have studied miRNAs which sensitize cells to the PARP inhibitor Olaparib using a large miRNA mimic screening, but did not identify *hsa-miR-182-5p* as involved in drug sensitivity [65]. It is tempting to speculate that different miRNAs might play a role in the action of different PARP inhibitors, but the genetic makeup of studied cell lines/models might contribute [65]. Since our study contained a large number of cell lines we reduced the influence of the genetic makeup on the identified miRNA-drug association favoring a more general link with Veliparib response. In our study *hsa-miR-182-5p* was associated with a large number of pathways, including the pathway “Cell cycle G1-S check-point” (see **Table 2**), which showed overall a downregulation. It remains, however, unclear how the downregulation of this cell cycle checkpoint might exactly contribute to drug resistance, although cell cycle changes have been earlier found as a drug resistance mechanism for PARP inhibitors [63].

The drug Tivantinib targets the c-MET kinase and prevents it from downstream signaling [66]. In our screening we found 3 miRNAs significantly associated with Tivantinib drug response. *Hsa-let-7d-5p* and *hsa-miR-18a-5p* were associated with drug sensitivity and *hsa-miR-637* with drug resistance. Interestingly, others have found that *hsa-miR-637* expression leads to downregulation of *STAT3* activity in hepatocellular carcinoma cells [67] and STAT3 is one of the downstream activated proteins of c-MET activity [68]. Furthermore, HGF-c-MET signaling also leads to activation of the PI3K/AKT pathway [69] and *AKT1* has been identified as a direct target of *hsa-miR-637* in pancreatic ductal adenocarcinoma cells [70]. One can speculate that the inactivation of c-MET effector pathways (through *AKT1* and *STAT3*) through this miRNA characterizes cells which rely on different pathways for growth and survival and therefore identifies cell lines inherently resistant to c-MET inhibitors.

Regarding Tivantinib sensitivity, one of the identified miRNAs was *hsa-let-7d-5p*. This miRNA has been shown to be downregulated by *STAT3* in breast cancer cells [71]. This contradicts the above hypothesis that Tivantinib-sensitive cells are characterized by active *STAT3* and high *hsa-let-7d-5p* expression and Tivantinib-resistant cells have inactive/low *STAT3* and high *hsa-miR-637* expression. Nevertheless, we also observed a negative correlation between *hsa-miR-637* and *hsa-let-7d-5p* in line with their opposing association with drug sensitivity. Other factors might play an additional role here. The last Tivantinib-associated miRNA, *hsa-miR-18a-5p*, associated with drug sensitivity, did not show significantly correlated expression with *hsa-let-7d-5p*. Others have found that *hsa-miR-18a-5p* targets *PIAS3* directly and in this way causes an increase in *STAT3* transcriptional activity in gastric cancer cell lines [72] fitting with the hypothesis for *hsa-miR-637*.

For the proteasome inhibitor Bortezomib [53] several resistance mechanisms have been found. Thioredoxin reductase 1 (HGNC symbol: *TXNRD1*) upregulation has been linked to Bortezomib resistance via upregulation of NF-κB-regulated genes in myeloma cells [73], as well as the upregulation of heat shock proteins and related genes in several cancer types [74]. Further resistance mechanisms are upregulation of proteasome subunits or increased proteasome activity which were observed in mesothelioma and myeloma [74]. Upregulation of the aggresome/autophagy pathway (both in myeloma), and constitutive NF-κB or AKT signaling in multiple myeloma have also been observed [75]. *Hsa-let-7a-5p* was associated with Bortezomib sensitivity in our study. This miRNA has been shown to target KBRAS2 (*NKIRAS2)*, an inhibitor of NF-κB signaling and seems to be itself upregulated upon NF-κB signaling in human macrophages [76]. *Hsa-let-7a-5p* targets furthermore a negative regulator of NF-κB signaling, *TNFAIP3*, in HEK293T cells [77]. NF-κB pathway inhibition is one of the effects of Bortezomib treatment [74], but generally activation of NF-κB signaling has been implicated in Bortezomib resistance [75]. Interestingly, there has been one report stating that upregulation of NF-κB signaling due to treatment with Lapatinib sensitized cells to Bortezomib in triple-negative breast cancer [78]. It remains therefore unclear whether upregulation of NF-κB signaling aids to sensitize cells to Bortezomib or increases drug resistance in breast cancer and whether *hsa-let-7a-5p* increases Bortezomib sensitivity via downregulation of *NKIRAS2* and *TNFAIP3*.

Sensitivity to the FGFR inhibitor JNJ-707 [40] was associated with *hsa-miR-135a-3p*, however, so far very little is known about this miRNA and unfortunately no information supporting the association with this drug could be found.

For the histone deacetylase (HDAC) inhibitor Panobinostat [55] several resistance mechanisms have been observed such as overexpression of the anti-apoptotic protein Bcl-2 in cutaneous T-cell lymphoma patients [79]. Of interest is also the observation that Panobinostat treatment led to activation of NF-κB signaling in leukemic cells and the blockage of this signaling increased sensitivity to the drug [80]. In our study, we found that the expression of *hsa-miR-185-3p* was associated with sensitivity to Panobinostat. However, little is known so far about the functions of this miRNA and there are no known targets which could explain how this miRNA might sensitize cells to Panobinostat.

For the farnesyltransferase inhibitor Tipifarnib [56], there are a few reports on resistance mechanisms. In previously untreated AML patients a 2-gene-classifier has been found which can predict response to Tipifarnib [81]. Patients responding to treatment are characterized by high expression of *RASGRP1*, a guanine nucleotide exchange factor which can activate RAS and low expression of *APTX*, a protein involved in DNA excision repair [81]. One of the properties Tipifarnib has is the inhibition of RAS farnesylation [82] and the upregulation of *RASGRP1* might characterize cancer cells which rely on RAS signaling. In our study *hsa-miR-629-5p* was associated with Tipifarnib resistance, however, based on the current knowledge about this miRNA, it is unclear if there is a direct mechanistic link between the observed drug resistance and the miRNA and how this miRNA might influence drug response.

Among the miRNA-drug associations where subtype played a role, *hsa-miR-23a-3p* was expressed lower in luminal cell lines. Others have found that this miRNA targets the progesterone receptor [83] and seems to be downregulated by estradiol [84], which could support preferential expression of this miRNA in triple-negative cell lines. *Hsa-miR-338-3p* was expressed lower in the basal subtype and higher in the luminal subtype. The basal subtype in cell lines is characterized by *EGFR* expression in contrast to the luminal subtype which typically lacks expression of this protein[43]. Interestingly it has been shown that *EGFR* expression downregulates *hsa-miR-338-3p* expression[85], matching our observations on the breast cancer cell lines.

## 5. Conclusions

We were able to identify several miRNAs associated with drug resistance or drug sensitivity in our large panel of breast cancer cell lines. Several of the miRNAs found in our screen have been linked to pathways targeted by the drugs or genes involved in drug-resistance mechanisms. Next to those associations, we also identified a number of miRNAs, which have not been researched much in the context of drug sensitivity/resistance so far but may hold great potential once more is known about their biology.

Besides identifying miRNAs associated with drugs already used in the clinic we also identified miRNAs associated with several new anticancer agents, which are expected to enter the clinic in the coming years. Since biomarkers can help in discriminating those patients which will respond better to therapy and miRNAs are well measurable, further research into this topic will be of great value and might potentially validate these miRNAs as biomarkers.

In conclusion, our hypothesis-generating study suggests that miRNAs could be used as predictors of drug response and once independently validated and/or experimentally confirmed holds great potential for an application in the clinic.

## Supporting information

S1 Table MiRNA expression data for the cell line OCUB-M. MiRNA expression data for the cell line OCUB-M is given including the respective probe ID for each miRNA assessed.
(XLSX)

S2 Table Annotation information for the miRNAs associated with drug response. The former and current miRNA nomenclature is given, together with the miRBase [45] MIMAT identifier and the Exiqon oligonucleotide probe ID.
(XLSX)

S3 Table Overview of all drugs and the employed analysis method. All drugs used in this study are listed with their respective type of analysis. The IC_50_ values and profiles of all drugs are available in the supplemental data of a previous publication [40].
(XLSX)

S4 Table Multivariate analysis with molecular breast cancer subtype. MiRNAs significantly associated with molecular breast cancer subtypes. Breast cancer subtypes assessed include luminal, basal and normal-like subtype. MiRNAs which are not associated with any of the three subtypes are printed in bold.
(XLSX)

S5 Table MiRNAs associated with Tivantinib. Spearman correlation coefficients among the different miRNAs are listed and the significant associations are highlighted in orange or blue depending on the correlation type. Orange = significant negative association, blue = significant positive association. Furthermore, all p-values of the associations are given.
(XLSX)

S6 Table MiRNAs associated with Docetaxel. Spearman correlation coefficients among the different miRNAs are listed and the significant associations are highlighted in orange or blue depending on the correlation type. Orange = significant negative association, blue = significant positive association. Furthermore, all p-values of the associations are given.
(XLSX)

S7 Table MiRNAs associated with Paclitaxel. Spearman correlation coefficients among the different miRNAs are listed and the significant associations are highlighted in orange or blue depending on the correlation type. Orange = significant negative association, blue = significant positive association. Furthermore, all p-values of the associations are given.
(XLSX)

S8 Table MiRNA expression and IC_50_ values per cell line. MiRNA expression data for the significantly associated miRNAs, not influenced by subtype are given per cell line. The IC_50_ values of the respective associated drug are given as well per cell line. Cell lines are color-coded based on their subtype: green = luminal, orange = basal, black = normal-like. MiRNA expression values and IC_50_ values are colored in a red-green color range with highest values in red and lowest in green.
(XLSX)
